# Fine scale diversity in the lava: genetic and phenotypic diversity in small populations of Arctic charr *Salvelinus alpinus*

**DOI:** 10.1186/s12862-024-02232-3

**Published:** 2024-04-15

**Authors:** Camille A. Leblanc, Katja Räsänen, Michael Morrissey, Skúli Skúlason, Moira Ferguson, Bjarni K. Kristjánsson

**Affiliations:** 1 Department of Aquaculture and Fish Biology, Hólar University, Sauðárkrókur, Iceland; 2 Department of Biology and Environmental Science, University of Jyväskylä, Jyväskylä, Finland; 3 School of Biology, University of Saint Andrews, Saint Andrews, UK; 4 Icelandic Museum of Natural History, Reykjavik, Iceland; 5 Department of Integrative Biology, University of Guelph, Guelph, Ontario Canada

**Keywords:** Small population size, Neutral processes, Drift, Phenotypic variation, Morphology, Lava cave, Fish movement

## Abstract

**Background:**

A major goal in evolutionary biology is to understand the processes underlying phenotypic variation in nature. Commonly, studies have focused on large interconnected populations or populations found along strong environmental gradients. However, studies on small fragmented populations can give strong insight into evolutionary processes in relation to discrete ecological factors. Evolution in small populations is believed to be dominated by stochastic processes, but recent work shows that small populations can also display adaptive phenotypic variation, through for example plasticity and rapid adaptive evolution. Such evolution takes place even though there are strong signs of historical bottlenecks and genetic drift. Here we studied 24 small populations of the freshwater fish Arctic charr (*Salvelinus alpinus*) found in groundwater filled lava caves. Those populations were found within a few km2-area with no apparent water connections between them. We studied the relative contribution of neutral versus non-neutral evolutionary processes in shaping phenotypic divergence, by contrasting patterns of phenotypic and neutral genetic divergence across populations in relation to environmental measurements. This allowed us to model the proportion of phenotypic variance explained by the environment, taking in to account the observed neutral genetic structure.

**Results:**

These populations originated from the nearby Lake Mývatn, and showed small population sizes with low genetic diversity. Phenotypic variation was mostly correlated with neutral genetic diversity with only a small environmental effect.

**Conclusions:**

Phenotypic diversity in these cave populations appears to be largely the product of neutral processes, fitting the classical evolutionary expectations. However, the fact that neutral processes did not explain fully the phenotypic patterns suggests that further studies can increase our understanding on how neutral evolutionary processes can interact with other forces of selection at early stages of divergence. The accessibility of these populations has provided the opportunity for long-term monitoring of individual fish, allowing tracking how the environment can influence phenotypic and genetic divergence for shaping and maintaining diversity in small populations. Such studies are important, especially in freshwater, as habitat alteration is commonly breaking populations into smaller units, which may or may not be viable.

**Supplementary Information:**

The online version contains supplementary material available at 10.1186/s12862-024-02232-3.

## Background

A major goal in evolutionary biology is to understand the processes underlying phenotypic variation in nature. The geographic distribution of phenotypic variation within and between populations is a product of the complex interplay between natural selection and neutral evolutionary processes (such as drift and bottleneck), acting on historical and contemporary timescales. The role of natural selection in local adaptation is often inferred through correlations between phenotype and current ecological conditions [1, 2]. However, for organisms sampled in the wild, deviations of phenotypic divergence from neutral genetic divergence, and associations with environmental divergence, could also arise from phenotypic plasticity [3, 4]. Phenotypic plasticity can be adaptive, has a genetic basis [5], and may promote rapid local adaptation [6]. It is thus important when searching for evidence for local adaptation to take into account the role of historical evolutionary processes, such as bottlenecks, genetic drift or adaptation, on contemporary traits variation [7].

The study of fragmented or small populations of wild organisms has allowed the disentangling of the influence of drift, gene flow and geographic isolation in shaping phenotypic divergence (e.g. [8]). In archipelagos and islands, research has shown how the loss of genetic connectivity can rapidly result in strong neutral genetic structuring [9]. In such isolated systems the dispersal ability of organisms is reduced, potentially causing a rapid reduction of gene flow and an increase in drift-mediated divergence [9]. A shift in the relative role of different evolutionary mechanisms is reflected by a typically positive relationship between neutral genetic diversity and population size (e.g. [8, 10]) that is only detectable if populations have been isolated for a number of generations. A less extreme scenario, corresponding to recent colonization event(s) or in a system where both gene flow and drift are equally shaping genetic diversity is reflected by patterns of isolation-by-distance [9, 11, 12], where geographically close populations are more genetically similar to each other than those further apart.

In comparison to larger populations, small populations are more influenced by demographic and environmental stochasticity [13–16]. Over time, small populations are expected to show increased rates of genetic drift leading to reduced genetic variation and lower adaptive potential [17]. However, the relationship between genetic variation and population size is unclear [18], and there appears to be no simple relationship between population size and the amount of additive genetic variation for fitness related traits among populations (morphological and behavioural traits), which is important for evolutionary responses (e.g. [19, 20]).

Teasing apart the evolutionary mechanisms shaping the genetics and phenotypes of small populations, at small spatial scales, is important but rarely done (but see [19–23]). Small populations are also expected to experience higher levels of inbreeding (as often shown by higher level of homozygosity [13, 24]) than larger populations, which combined with stronger genetic drift may result in more frequent fixation of deleterious alleles (e.g. [24]). Inbred individuals can have overall lower fitness (lower survival and/or lower reproductive success), and the relative effect of inbreeding on reducing fitness is stronger in small populations [19]. In addition, the genetic composition of the individuals forming founding population(s) at colonization, and the subsequent random variation in reproduction and survival among these individuals are crucial components for the evolution and the future of those small populations [25].

Freshwater fishes are powerful model systems for studying eco-evolutionary processes in small populations due to their geographically partitioned phenotypic and genotypic variation over multiple scales [26, 27]. In particular, northern freshwater fishes such as salmonids, show remarkable phenotypic variation across their geographic ranges following their recent recolonization into diverse habitats from glacial refuges [27, 28]. Local adaptation of fishes appears to be relatively common and is detectable at a variety of spatial scales from a few to thousands of km [18, 22, 29–31]. The availability of variable ecological resources is thought to be the main driver of adaptive divergence in freshwater fishes [26], with the genus *Salvelinus* a prime example of such diversity (reviewed in [29]).

Arctic charr (*Salvelinus alpinus*) shows marked morphological diversity within and among habitats throughout its range [29, 30]. Icelandic populations of Arctic charr are thought to have arisen from rapid postglacial recolonization from a single anadromous charr lineage, with subsequent restriction(s) of gene flow [31]. A prominent feature of diversity of Arctic charr is the frequent occurrence of “small benthic” phenotypes that live in relatively shallow water, often where groundwater emerges into springs in lava fields [32, 33]. These small benthic charrs are characterized by small size, large fins, robust body shape, a sub-terminal mouth and a dark coloration [33, 34]. Genetic studies suggest that the small benthic phenotype has evolved repeatedly in separate locations across Iceland [35].

In this study, we assessed the relative contribution of neutral versus non-neutral evolutionary processes in shaping phenotypic divergence across 24 small populations of *S. alpinus* inhabiting groundwater fed lava caves in Iceland. We contrasted patterns of phenotypic and neutral genetic divergence across populations located within a few km^2^. We combined population genetics tools, repeated sampling of individuals (mark-recapture) to estimate population size, as well as geometric morphometrics and environmental measurements, to estimate the relative contribution of neutral processes vs. non-neutral processes to phenotypic divergence. First, we used nine neutral microsatellite markers to infer population genetic structure arising from gene flow and drift. We tested for the effects of historical gene flow by determining if physically connected populations (based on fish movement patterns) are genetically more similar than those where no migrants have been observed. Second, we inferred phenotypic structure by characterizing variation in body and head shape, which are trophic traits linked to fitness. Third, we assessed if the spatial pattern of phenotypic variation corresponds to the neutral patterns of genetic diversity. We used two complementary analytical methods: we tested for an association between neutral genetic distances and phenotypic distances across populations, and we partitioned the amount of variation in phenotypic traits among populations into components. Those associated with environmental features (indicative of natural selection), neutral genetic structure and unexplained variation. We predicted that if natural selection and/or phenotypic plasticity have influenced phenotypic diversity, spatial patterns of phenotypic variation will deviate from neutral genetic patterns. Moreover, variance in morphology explained by the environment, when the neutral genetic structure is taken into account, would strongly suggest a role for plasticity and/or natural selection for the phenotypic traits shown to diverge.

## Results

### Cave features, physical connectivity and census population size

**Fig. 1:**
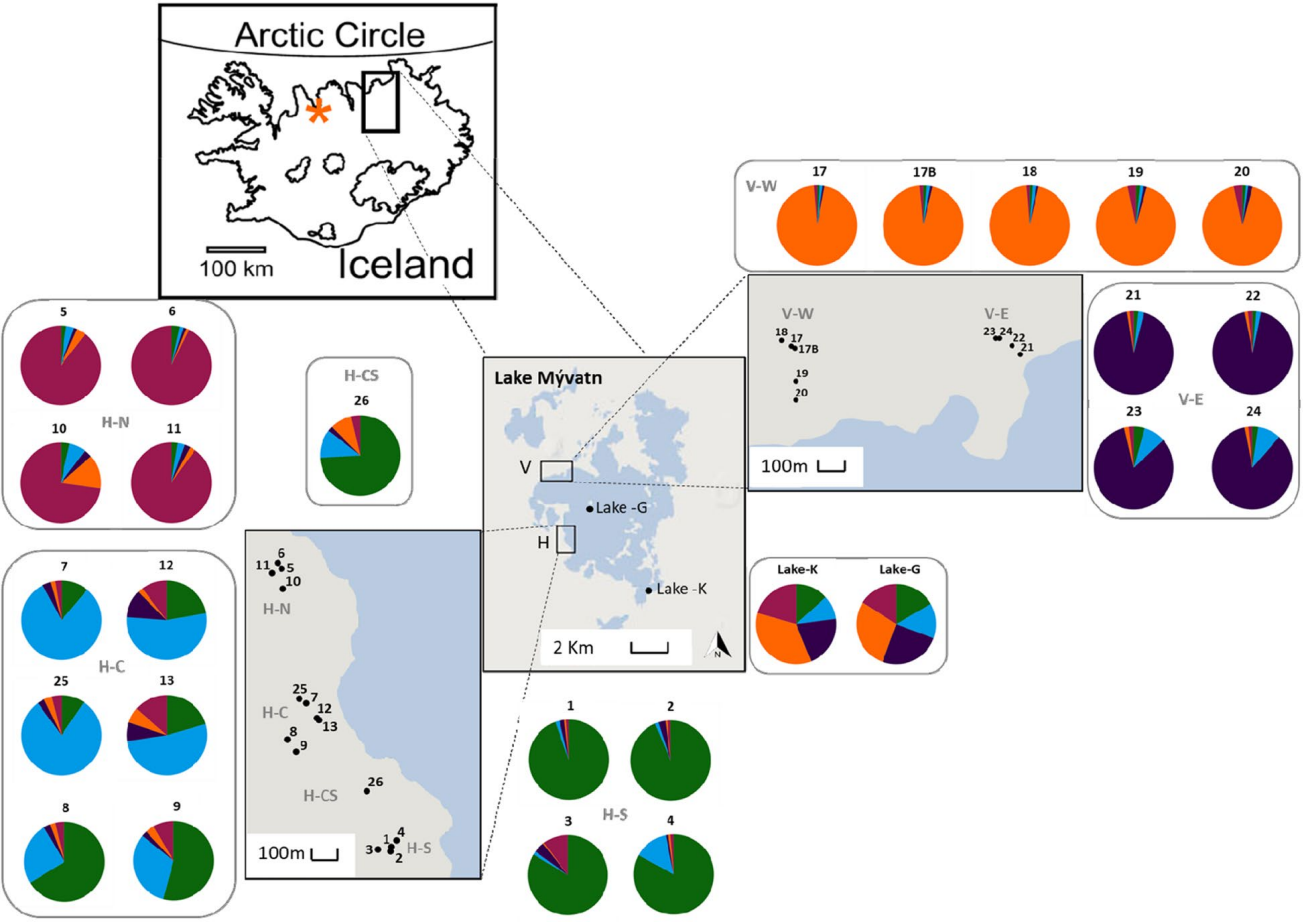
Location of 24 cave populations of small Arctic charr (*Salvelinus alpinus*) in the Vindbelgur and Haganes areas, Lake Mývatn northern Iceland. The caves in the Vindbelgur area (V) are clustered into western and eastern locations (W = west; E = east) while those in Haganes (H) can be subdivided into northern, central and southern clusters of caves (N = north; C = central; S = south). One cave (cave 26) is positioned between the H-C and H-S subareas. Lake populations are referred as the generalist (Lake-G) and the Krús morph (Lake-K). Pie charts indicate proportion of individuals affiliated with each genetic cluster according to results from STRUCTURE (K = 5). The contour of lake Mývatn was obtained and modified from the OpenStreet map contributors and the GIS user community. The map of Iceland was obtained and modified from Einarsson et al. 2004

**Fig. 2:**
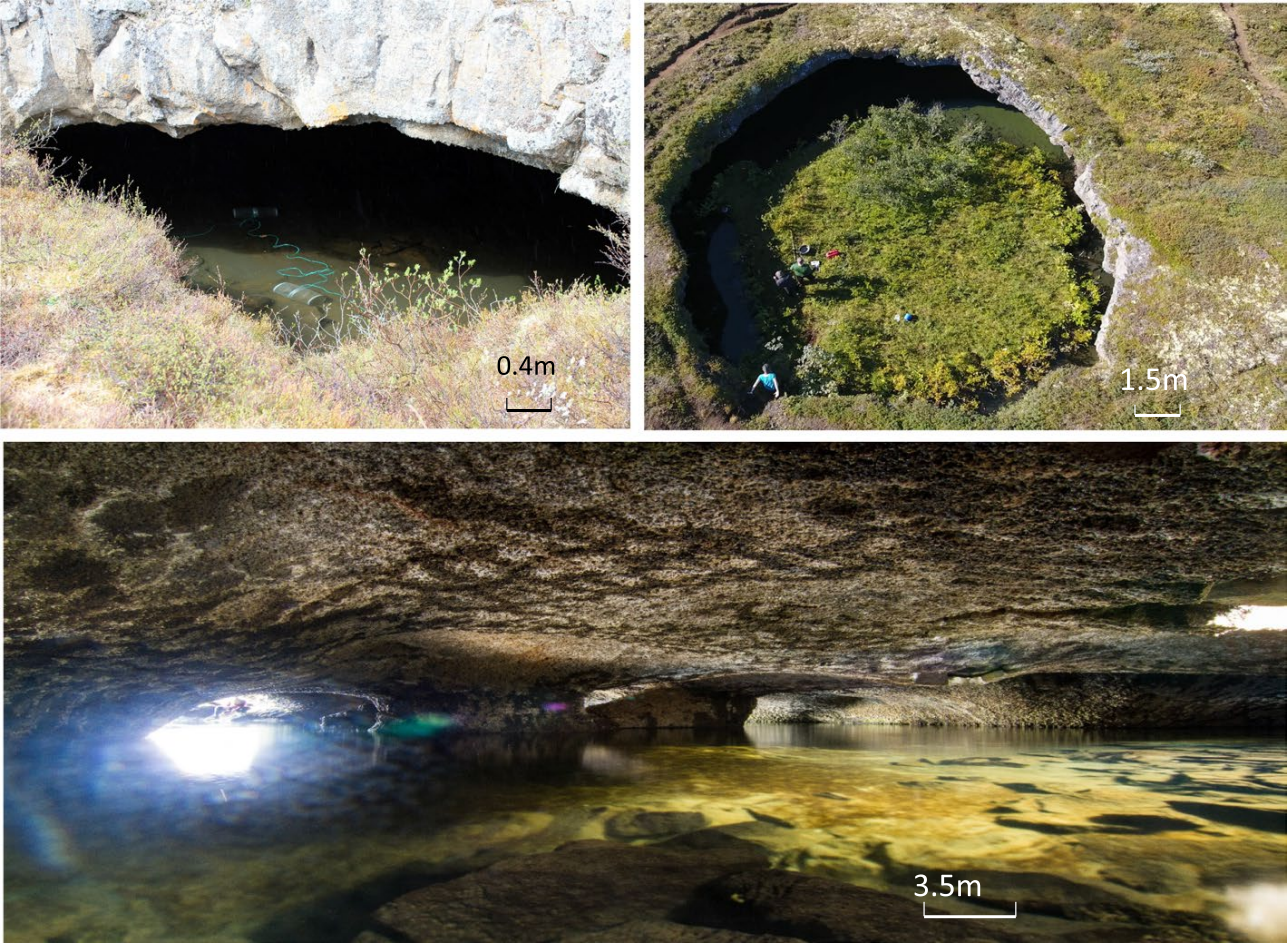
Lava caves filled with groundwater near Lake Mývatn, which contain small Arctic charr(*Salvelinus alpinus*). Caves of different sizes and spatial complexity are shown to display the diversity of lava caves present in this area. From top to bottom: main opening of cave 1 in the South of the Haganes area, with unbaited minnow traps laid to catch fish; drone picture of the main circular opening of the largest cave (cave 25) found in the center of Haganes area; view inside a large and complex cave (four openings, cave 5) found in the North of the Haganes area, lava rocks can be seen close to the openings and then mud cover the hard bottom towards the center of the cave. (Photo credits from left to right: Camille Leblanc, Árni Einarsson, Anup Gurung)

The 24 caves are located 57 to 500 m from the lake and vary in many ecological aspects (Figs. 1 and 2). The number of openings ranged from one to seven per cave, and the water area exposed to sky (i.e. area of openings) ranged from 0 m^2^ to 37.45 m^2^ per cave. Note that the zero values indicate that the terrestrial habitat is either covered by the lava ceiling (two caves), or there is no exposure to the sky (three caves). The size of the openings varied among caves, but was independent from the water area exposed to the sky. Average annual water temperature across all caves was 5.52 ± 0.7 °C (mean ± SD), and water temperature varied from 4.83 ± 0.1 °C in winter (September to May) to 6.21 ± 0.1 °C in summer (June to August).

Caves’ population size (*Nc*) ranged from 11 to 550 individuals based on mark-recapture data (Appendix 1). Movement of fish was observed between six pairs of caves (i.e. individuals tagged in one cave were recaptured in another cave indicating underground connectedness of these caves). The number of migrants in these six pairs of caves varied from one to 19 (mean ± SD = 6.7 ±; 6.34; median = 4.5; Appendix 1).

### Genetic diversity

Each of the cave populations showed relatively modest levels of genetic variation at the nine microsatellite loci and had 2–4 alleles per locus, with very few private alleles (Table 1). The lake samples were more genetically variable and contained all of the alleles seen in the caves, highly suggesting that the cave populations originate from the lake population. There were few deviations from HWE expectations within populations and the number was lower than expected by chance alone (data not shown). Only a single population, and none of the loci, showed consistent deviations from HWE. The one significant *F*_*IS*_ value was detected for cave 4, which had a very small sample size (*N* = 17). Highly significant differences in allele frequencies were detected in the vast majority of pairwise comparisons among populations, based on Fisher’s exact test after Bonferroni correction (only 13 out of 325 comparisons were not significant, Appendix 2). Similarly, multi-locus pairwise *F*_*ST*_ values were relatively high, and the 95% CI only included zero in the 13 cases where allele frequencies did not differ significantly (Appendix 2). Apparent cases of genetic homogeneity occurred between caves in close geographic proximity (Fig. 1; Appendix 2), and for pairs of caves where fish tagged in one cave had been recaptured in the other.

**Table 1:** Neutral genetic diversity at nine microsatellite loci and pairwise differentiation in Arctic charr from lava caves

*Area*	*Cluster*	*Caves*	*Latitude*	*Longitude*	*N*	*N*_*A*_ *± SD*	*Ar*	*P Ar*	*H*_*O*_	*H*_*E*_	*Fis*	*Null Alleles*
H-S	1	1	65°34′802”	17°03′032”	26	2.11±0.60	1.95	0	0.31	0.31	0.005	–
H-S	1	2	65°34′794”	17°03′035”	30	2.11±0.78	1.86	0.01	0.27	0.32	0.127	OMM1236/ OMM5146
H-S	1	3	65°34′796”	17°03′087”	15	2.22±0.83	2.02	0.01	0.25	0.26	0.033	–
H-S	1	4	65°34′812”	17°03′012”	17	1.89±0.60	1.83	0	0.49	0.33	-0.515^a^	–
H-N	5	5	65°35′278”	17°03′487”	56	3.33±2.06	2.78	0	0.49	0.51	0.038	BX890355/Omi179tuf
H-N	5	6	65°35′289”	17°03′502”	15	2.56±1.24	2.38	0	0.42	0.43	0.027	–
H-C	2	7	65°35′048”	17°03′385”	78	4.11±2.80	2.87	0.07	0.46	0.44	−0.044	–
H-C	2	8	65°34′986”	17°03′462”	9	2.78±0.97	2.75	0	0.53	0.48	0.109	–
H-C	2	9	65°34′965”	17°03′427”	21	3.56±1.88	3.02	0	0.51	0.51	−0.010	–
H-N	5	10	65°35′244”	17°03′481”	30	3.11±1.76	2.7	0	0.53	0.51	−0.032	–
H-N	5	11	65°35′271”	17°03′528”	48	3.33±1.58	2.82	0.02	0.48	0.51	0.053	BX890355/OMM1302/OMM1329
H-C	2	12	65°35′023”	17°03′337”	42	3.22±1.64	2.81	0.02	0.50	0.50	−0.011	–
H-C	2	13	65°35′021”	17°03′334”	14	3.33±1.50	2.96	0.06	0.55	0.55	−0.016	OMM5151
V-W	4	17	65°37′018”	17°04′262”	10	2.22±0.83	2.2	0	0.41	0.38	−0.076	–
V-W	4	17b	65°37′014”	17°04′243”	16	2.22±0.83	2.19	0	0.40	0.39	−0.039	–
V-W	4	18	65°37′030”	17°04′309”	60	2.56±0.88	2.27	0	0.40	0.38	−0.048	–
V-W r	4	19	65°36′946”	17°04′240”	26	2.89±1.05	2.66	0.01	0.47	0.45	−0.049	–
V-W	4	20	65°36′908”	17°04′239”	43	3.00±1.12	2.64	0.01	0.42	0.45	0.066	–
V-E	3	21	65°37′001”	17°03′123”	35	3.11±1.54	2.58	0.07	0.50	0.47	−0.077	–
V-E	3	22	65°37′021”	17°03′166”	71	3.78±2.17	2.69	0.02	0.44	0.47	0.049	OMM5151/OMM5146/OMM1211
V-E	3	23	65°37′034”	17°03′246”	38	3.00±1.58	2.43	0.02	0.38	0.38	−0.001	–
V-E	3	24	65°37′034”	17°03′229”	22	3.11±1.54	2.72	0.06	0.48	0.50	0.030	OMM5146
H-C	2	25	65°35′055”	17°03′416”	182	4.33±3.16	2.87	0.05	0.44	0.46	0.040	OMM1211
H-CS	1	26	65°34′896”	17°03′134”	69	3.44±1.24	2.65	0.05	0.43	0.41	−0.047	BX890355
L	Lake	Generalist			50	6.56±4.50	3.84	0.44	0.55	0.59	0.082	OMM5151/OMM1329
L	Lake	Krús	65°33′052”	16°56′038”	49	5.33 3.20	3.78	0.23	0.56	0.61	0.083	OMM1211/ OMM5146

Fish were caught in the Haganes (H) and Vindbelgur (V) geographical areas near Lake Mývatn, Iceland. Samples of benthic (Krús) and generalist charr were also sampled from Lake Mývatn (L). The coordinates of each sampling site (cave or lake), number of individuals genotyped (N), number of alleles (N_A_ ± SD), allelic richness (Ar, 16 genes), private allele richness (P Ar, 16 genes), observed (H_o_) and expected heterozygosity (H_e_), fixation index (F_IS_) and Structure cluster membership (see text for details) are shown. ^a^ Significant deviation from Hardy-Weinburg proportions

**Fig. 3:**
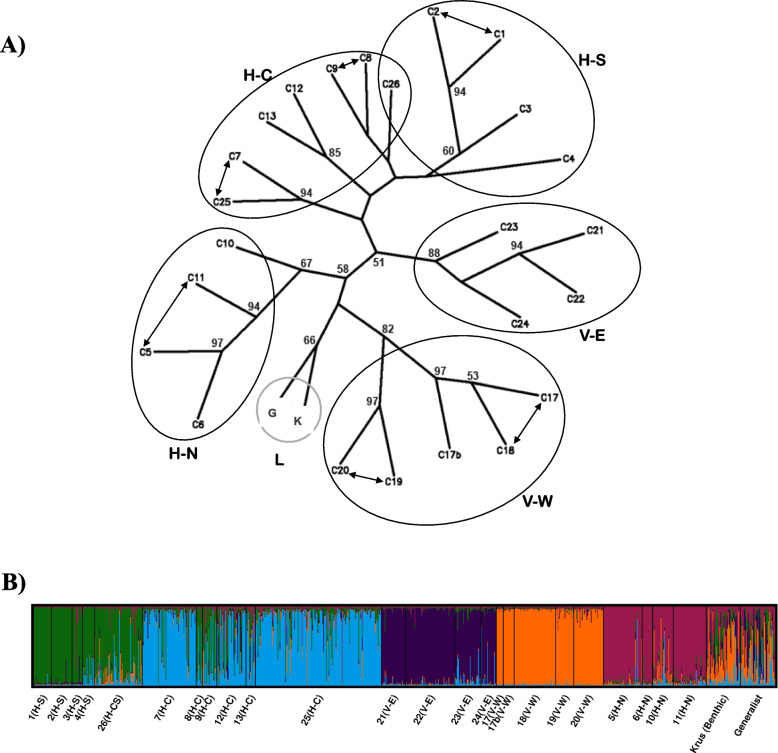
Neighbour joining phenogram and Bayesian clustering analysis for 24 caves and 2 lake populations of Arctic charr. **a** Neighbour joining phenogram based on Cavalli-Sforza and Edward’s chord distance (D_ce_) showing relationships among cave populations (C) and lake populations (L). Only nodes with greater than 50% bootstrap support are shown. Double arrows indicate movements of tagged fish between two caves; pairwise Fst and Dest values were not significant in these cases (Appendix 2). **b** Bayesian clustering analysis using STRUCTURE for *K* = 5. The probability of assignment for each individual to each cluster is shown in a vertical bar. Labels correspond to the population identifier, the geographic area (V=Vindbelgur; H=Haganes) where the population is located and the subarea within that area (W = west; E = east; N = north; C = central; S = south). Each color corresponds to a distinct genetic cluster (Fig. 1)

The neighbour joining phenogram based on *Dce* values (Appendix 1) detected some geographically based structuring that was more evident at the scale of sub-area (e.g. N, C, S sub-areas within H and E, W sub-areas V) than at the larger scale (i.e. H vs V areas; Fig. 3A). To large extent there was good bootstrap support for affinity among the populations within each of the V-E (88%), V-W (82%), H-N (67%), H-S (60%) subareas (excluding C4), but not for the H-C populations (< 60%). At the larger scale, the populations within each of the H and V areas did not cluster together (Fig. 3A). STRUCTURE analysis identified five genetic clusters among the cave populations (Appendix 3), showing some correspondence to the geographic subareas (Table 1, and Fig. 3B). Within each of the H-S, H-N, V-E and V-W subareas individuals had a high likelihood of being assigned to a single cluster, which was shared across populations within the subarea. For example, the fish captured from the four caves in the H-S subarea had the greatest likelihood of membership (> 80%) to cluster 1. Fish from other subareas (e.g. V-E versus V-W versus H-N versus H-S) had different cluster affinities with evidence of low levels of admixture (Fig. 3A). Fish from cave 26 located between the H-C and H-S subareas showed some evidence of admixture and the greatest affinity to fish from the H-S subarea (Fig. 3A). Fish from the lake - Krús and generalist - showed affinity to multiple clusters (indicated by the diversity of colors in Figs. 1 and 3 B), which may indicate that cavefish primarily originate from both lake morphs. There were subtle differences in allelic composition between the two morphs in the lake (Figs. 1 and 3, Appendix 1), indicating a sympatric divergence between them.

There was an overall positive relationship between genetic and geographic distance matrices (Mantel test, *R* = 0.39, *P* = 0.0001), suggesting a pattern of isolation by distance (IBD). However, this relationship was mostly caused by the distance of the caves to the lake. There was no support for IBD when controlling for the geographic areas in the test (H-V-lake; partial-mantel test, *R* = 0.10, *P* = 0.13).

### Morphological diversity

**Fig. 4:**
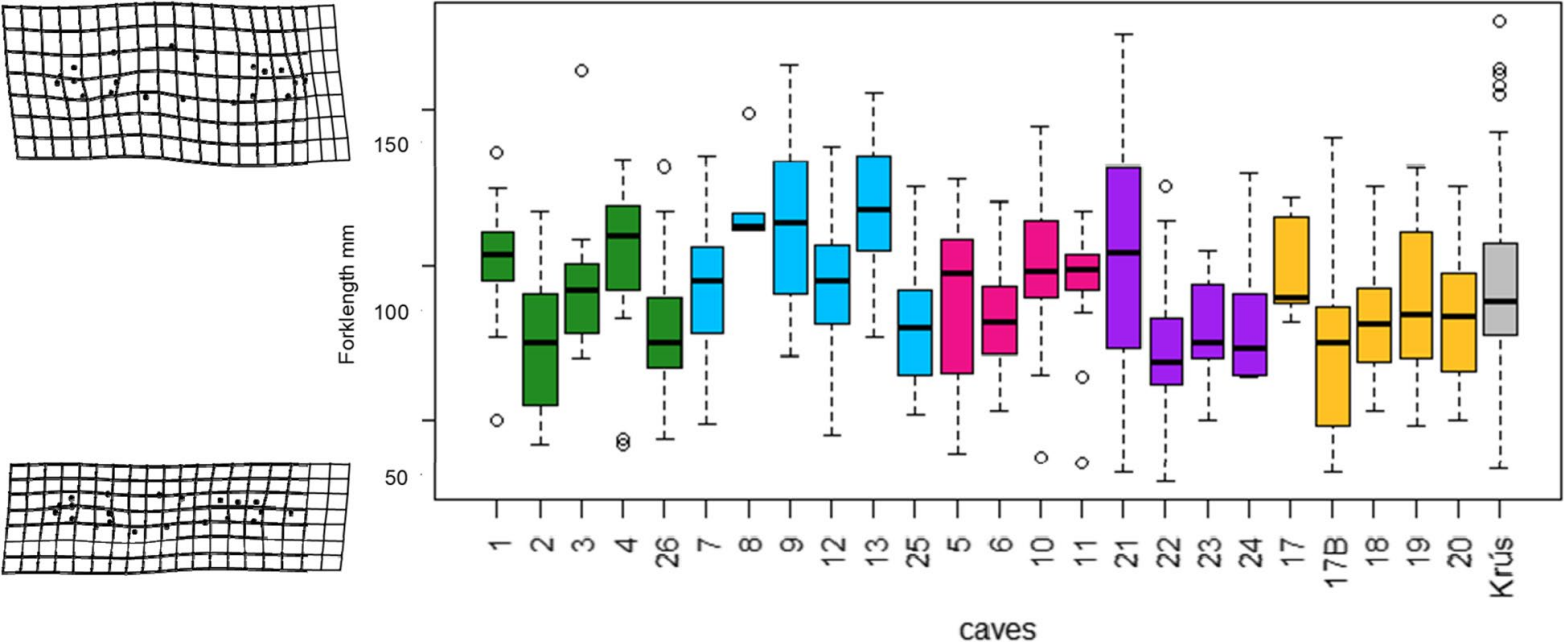
Variation in body size and body shape in 24 populations of Arctic charr (*Salvelinus alpinus*) found in lava caves, Iceland. Fork length in millimeters (y axis) was used as a measure of body size. Body shape of individuals was obtained from landmarks-based geometric morphometrics using Procrustes linear models. Thin plate splines deformation grids (surimposed to the y axis at both extremes) show the deviation from the overall consensus shape with a 2-time magnification. Total sample size per cave ranged from 5 individuals in cave 8 to 56 in cave 25. The charr morph from the lake is refered to as Krús. Each cave is colored according to its genetic clusters as per Fig. 1

**Fig. 5:**
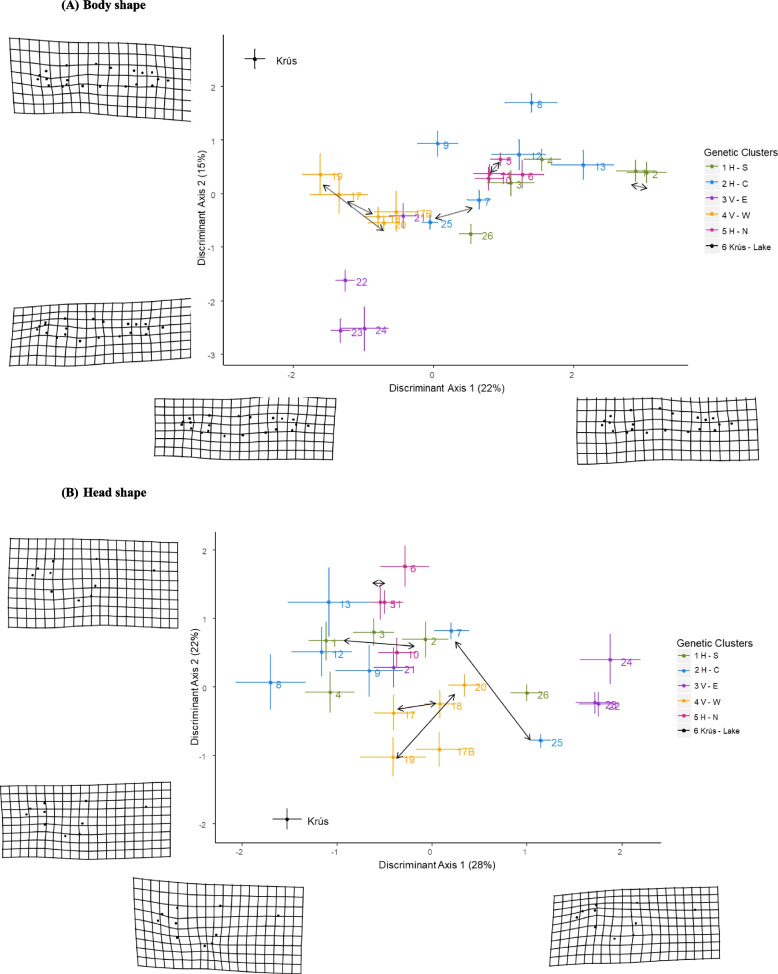
Discriminant Function analysis of phenotypic diversity among 24 populations of Arctic charr in lava caves. Phenotypic diversity was estimated as (**a**) body shape and (**b**) head shape using landmarks-based geometric morphometric of body morphology and discriminant function analysis. Each point represents the average body/head shape of fish in a given population. Numbers refer to the cave numbers as described in Table 1. The deformation grids show the average morphology of fish/head in a population with a 3-time magnification, at the extremes. The genetic cluster that the majority of fish in a population were assigned to is indicated by the color of the average body shape point (main geographic area: V=Vindbelgur; H=Haganes, and subarea W = west; E = east; N = north; C = central; S = south; see Table 1 and Fig. 3 for details). The morph from the lake is refered to as “Krús”. Double arrows indicate movements of tagged fish between the caves (i.e. migrants). Fish from connected caves (double arrows) showed variable similarities in body and head shape. For instance, fish from caves 1 and 2 (10 migrants) were phenotypically similar in body shape, but in other cases (caves 7 and 25 with 19 migrants) were very different. The differences between fish from caves 25 and 7 were even more marked for head shape

Fish size differed among the populations, both in centroid size (ANOVA, *F*_(24,493)_ = 45.1, *P* < 0.01) and in FL (ANOVA, *F*_(24,493)_ = 4.3, *P* < 0.01; Fig. 4). Body shape was moderately related to FL (Procrustes linear models: *F*_(1,517)_ = 70.99, *P* < 0.01, *R*^2^ = 0.12): smaller fish had narrower bodies, a longer caudal peduncle and a longer caudal fin (Fig. 4). Average body shape differed among the populations (F_(24,517)_ = 6.2, *P* < 0.01, R^2^ = 0.19; Fig. 5 a). However, whilst FL had a significant effect on body shape, this relationship differed among the populations (FL*population interaction: *F*_(24,517)_ = 2.2, *P* < 0.01, *R*^2^ = 0.07).

Both PCA and DFA were used to visualize body and head shape variation among populations. PCA results and description of shape variation can be found in the supplementary material (Appendix 4). In brief, most changes in body shape (Appendix 4A) were seen in body depth, with particular changes in the robustness of the head and the length of the caudal peduncle. Fish with the highest PC1_body_ scores had robust heads (deep and long) and shorter caudal peduncles. PC3_body_ reflected changes in overall body depth and length of the caudal peduncle, with the highest scores associated with a more streamlined body, longer caudal peduncle and a lower set eye (Appendix 4A). PC5_body_ depicted differences in the insertion of the opercula and the pectoral fin. PC2 _body_ and PC4 _body_ captured artefacts of the sample processing mostly up and down bending, and do not reflect morphological variation. They were thus not used in the analysis.

Examination of the morphological variation based on DFA revealed clear differences in body shape among populations. The first two axes of the DFA_body_ explained 37% of the total variability. The Krús morph was morphologically differentiated from all cave populations along both DF1_body_ and DF2_body_ (Fig. 5A), which may be linked to size differences. Populations from the V and H areas were differentiated along DF1_body_: fish from the V area had smaller head and thinner bodies than fish from the H area. There was some separation between populations V-E and V-W areas along DF2_body_, but the populations from the three subareas of H were not differentiated on either axis (Fig. 5A). Based on body shape, the accuracy of classification fish to their a priori cave population was between 39 and 100% (average 75%). 92% of the Krús were correctly classified.

Head size (ANOVA, F_(24,493)_ = 6.1, *P* < 0.001, R^2^ = 0.06) and head shape (F_(24,517)_ = 4.9, *P* < 0.01, R^2^ = 0.17) differed among populations Fig. 5B), but the relationship between centroid head size and head shape was different among populations (centroid size_head_*population interaction: *F*_(24,517)_ = 2.02, *P* < 0.01; R^2^ = 0.071).

The PCA of head shape across caves (Appendix 4B) revealed that most variation in the head was primarily associated with the length of the maxilla, the setting of the eye and the position of the operculum. PC1_head_, PC2_head_ and PC3_head_ explained 38, 15, and 12% of head shape variation, respectively. Fish with higher PC1_head_ scores had a longer maxilla and a bulkier snout with a more posterior insertion of the operculum, whereas fish with higher PC2_head_ scores had a shorter maxilla, and a smaller snout with posterior insertion of the operculum (Appendix 4B). Fish with higher PC3_head_ scores had a longer maxilla, a more posterior insertion of the dorsal fin and thinner head.

The first two axes of the DFA_head_ explained 50% of the total variability. Analysis of head shape produced similar result to that of the whole body: the two subareas of V were differentiated from each other along DF1_head_ and from the H populations along DF2_head_. Fish from the different H subareas did not differ in head morphology (Fig. 5B), and showed some similarities with fish from V-E (cave 21). Based on head shape, the accuracy of classification of individuals to their a priori population, was between 5 and 82% (average 42%) for the cave populations. 82% of the Krús were correctly classified. The distribution of scores along the DF1_head_ axis indicated that fish from caves in the VE area had shallower heads, higher set eyes and a wider operculum than other fish from the V area, or fish from the H area (Fig. 5). Fish with higher DF2_head_ scores had more robust heads (longer and deeper heads). The head of the Krús morph was morphologically differentiated from all cave populations: they had overall shorter but deeper heads. Overall, body and head shape of fish from V-E and the Krús (lake) differed from the rest of the cave populations.

Although PCA analyses had revealed no phenotypic structuring in accordance with the genetic or geographic clusters (Appendix 4), the DFA analysis showed that fish could be reassigned to their cave of origin based on subtle morphological differences. Pairwise morphological distances in body and head shape were not related to geographical distances (Mantel test: body R = 0.004, *P* = 0.43; head R = − 0.069, *P* = 0.81), even after accounting for geographical area (partial mantel test: body R = − 0.034, *P* = 0.59; head R = − 0.077, *P* = 0.69).

### The relative importance of genetic diversity and local environment in shaping phenotypes

As a first approach, we checked for a direct association between genetic and morphological distances as a potential indication of neutral processes in shaping phenotype. There was no association between pairwise morphological distances in body/head shape and genetic *Dst* (Mantel test: body R = − 0.024, *P* = 0.58; head R = − 0.094, *P* = 0.83), even after accounting for area (H-V-Lake; partial Mantel test: body R = 0.022, *P* = 0.55; head R = − 0.075, *P* = 0.69; Appendix 5).

**Fig. 6:**
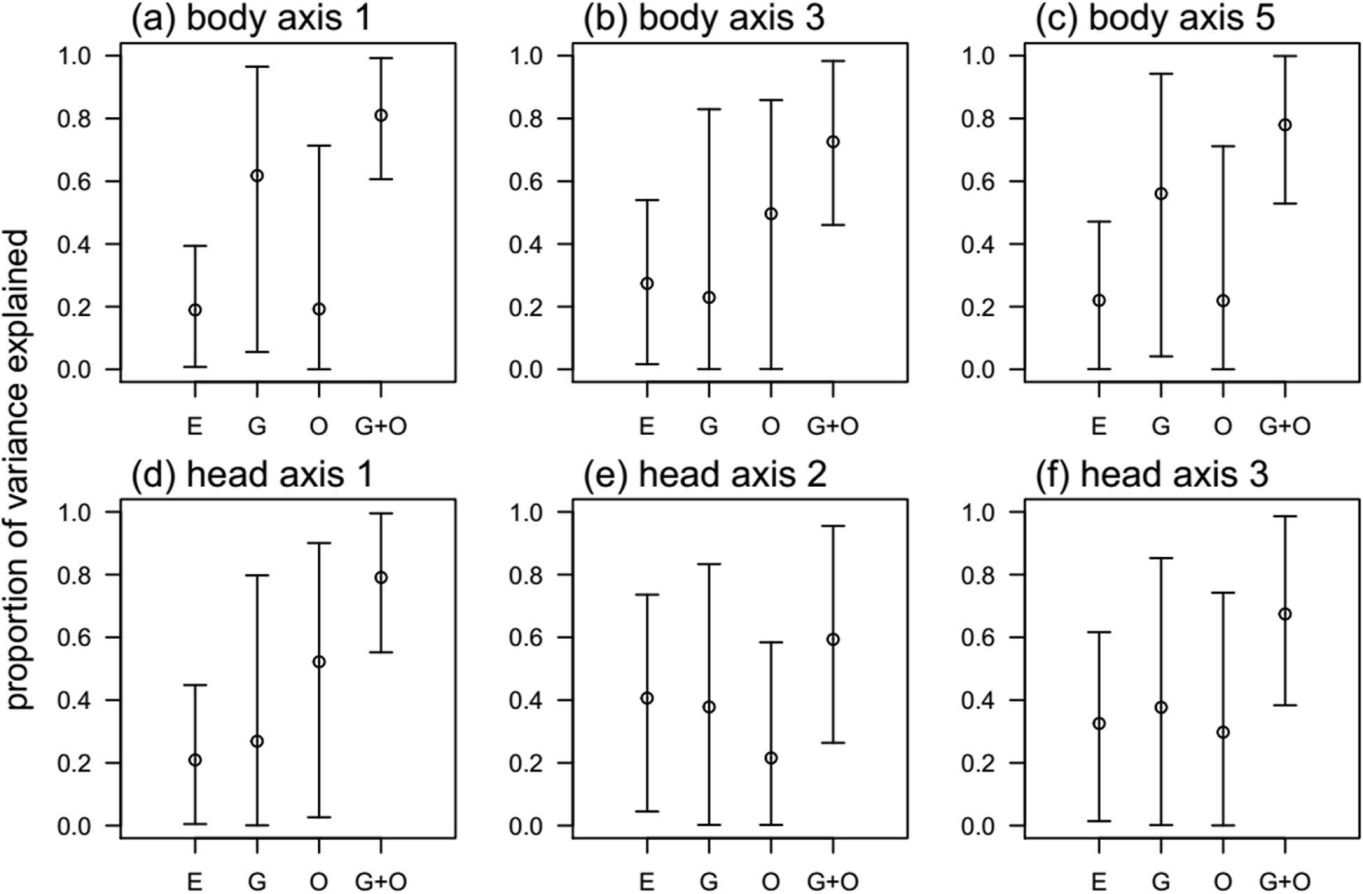
Proportions of among-caves variances in phenotypes accounted for by ecological variables, genetic distances, and not explained by either of those. Phenotypes were PC scores for body shape (PC1, 3, and 5, top row) and head shape (PC1, 2, and 3, bottom row), and genetic distances were Cavalli distance matrix (Dce; referred as G). Environmental variables (E) were the number of openings, the minimal distance to the lake and the annual average water temperature in each cave. Proportion of variance that was not explained by either of those refers to “others” (O)

The mixed model approach indicated that the proportion of variance in body shape, based on axes PC1_body_ and PC5_body_, was more associated with neutral genetic variation than with environmental or other factors (Fig. 6a-c). In contrast, most of the variance of PC3_body_ was not explained by neither the environmental variables nor the genetic structure. The among-cave partitioning of variance revealed that typically only a modest amount of variation in body shape among caves was explained by the environmental variables (Fig. 6), with point estimates of the proportion of variance explained ranging from about 20–40%.

Approximately 40% of the variance in head shape (PC2_head_ and PC3_head_) was explained by the environment (Fig. 6, e-f). Overall, some variance in the phenotype among caves was explained by genetic structure, but large uncertainties remained as 60–80% of variation among caves was unexplained (error bars in Fig. 6). Those uncertainties might be explained partly by modest sample sizes. While it is difficult to partition among-cave variation into components associated with genetic structure and “other”, there were nonetheless clear evidence for phenotypic variation at the among-cave level. The proportion of the total among-cave phenotypic variance in the PC traits (components associated with genetic structure and otherwise), as function of the total among-cave and within-cave (residual) variance, was different from zero in all cases (Appendix 6; repeatability was highest for PC1_body,_ PC3_body_ and PC1_head_). We report here only the partitioning of variance, but the fixed effects and random effects variances of the mixed model can be found in Appendix 7.

## Discussion

We tested for the relative contribution of neutral versus non-neutral evolutionary processes in shaping phenotypes of wild Arctic charr inhabiting 24 lava caves across a small spatial scale, and compared patterns of phenotypic and genetic variation to a nearby lake population. These populations harbour low genetic diversity, and show a pattern of isolation by distance (IBD) with the lake populations). The cave populations were most likely founded by fish from Lake Mývatn, although the colonization history remains unclear. We found patterns of morphological convergence and divergence in body and head shape among the populations. No relationship was found between the spatial patterns of phenotypic and neutral genetic variation and no correlation between genetic and morphological distances across populations. A statistical mixed modelling approach, testing for the relative contribution of neutral genetic processes versus influence of environment, showed that phenotypic variation was better associated with neutral genetic diversity with only a relatively small effect of the environment on fish head. These different approaches show that contemporary patterns of phenotypic diversity in these small fish populations is largely the product of neutral evolutionary processes, fitting with the classical theory of evolution in small populations [11, 14–16]. Whilst variation in body and head shape among populations was large, the modelling approach indicated that only a small proportion of this variation was under the influence of the environmental factors measured here. However, given the clear phenotypic divergence of Arctic charr in traits typically under strong selection (e.g. [34]) and/or diet induced plasticity, it is possible that environmental factors not measured here could underlie trait divergence among these populations.

### Genetic divergence

The distribution of neutral allelic variation within and among the cave populations relative to the lake samples, combined with fish movement data, indicates that founder effects, genetic drift and gene flow have contributed to contemporary patterns of genetic diversity, as is likely in small fragmented populations (e.g. [9]). The cave populations contain a subset of the allelic variation found in the two morphs of Arctic charr in the nearby Lake Mývatn which strongly suggests that the cave populations were founded from the lake populations, potentially via multiple colonization events and routes. This is indicated by stronger genetic structuring at the level of geographic subarea than at level of area. The data do not support a two-stage model, where fish dispersed into the V and H areas and then colonized the caves within all subareas within them. This prediction is further supported by the lack of IBD when geographic area is considered, although IBD is commonly observed when small populations originate from one source population [9, 11, 12].

The lower amount of genetic variation found in the cave populations, relative to the lake populations, is likely a signature of founder effects and/or genetic drift occurring after the obstruction of migration routes between the caves and the lake. It is likely that the caves were colonized by either flooding events, or through underground channels (e.g. [34]). Such underground water ways may have closed, by silt build-up in the cave ponds, or as a result of important water levels changes of Lake Mývatn [36]. There is evidence that gene flow (although limited) is still occurring among some of the caves. Fish in caves where movement is observed are genetically similar and cluster together. Further study on the system using different approach (i.e. SNPs) may reveal better the colonization history of the lava caves and identify genetic bottlenecks.

### Morphological divergence

Morphological divergence is well studied in fishes and classically characterised along various environmental gradients or between contrasting habitats (e.g. [37–40]). Lentic versus lotic freshwater habitats and related prey availability are often associated with intraspecific variation in body shape and head shape among fish populations, and benthic versus limnetic feeders diverge in head morphology (reviewed in [26]). In Arctic charr, it has been found that small benthic charr living in ponds have deeper bodies, shorter and wider caudal peduncles, and eat more chironomid larvae than fish from stream habitats [34]. These morphological differences have also been associated with differences in water conductivity and temperature as well as the roughness of the substrate. In our study, the environmental differences among the caves in the three proxies used (temperature, distance and cave openings) did not show any strong contrasts or gradients. However, body shape and head shape did differ among the cave populations. The differences were mostly seen in body depth and head size and shape, specifically in the maxilla and the snout of the fish which are associated with feeding in small benthic charr in Iceland [34]. Although subtle, those differences in body and head shape allowed a good classification of individual fish into their cave of origin. This indicates that within each cave fish have a defined morphology, which may be the results of local adaptation (see below) or clear plastic responses as commonly seen in Arctic charr [5]. The cave charr populations differ from other small benthic populations found in Iceland, in diet ecology, as their diet is highly diverse, but to large extent being taken from the surface of the pond [34], which may have a strong effect on head morphology. The importance of surface feeding may then differ among populations and is likely dependent on the size of the pond open to air. This need, however to be studied further.

The morphology of the fish was not correlated with geographic distances (i.e. fish found in caves close to each other did not have more similar morphology than fish found in more distant caves; Appendix 4). Moreover, the morphology of fish found in connected caves was not similar (Fig. 5). This may suggest that fish are somehow adapting (either genetically or through plasticity) to the cave they inhabit, and furthermore indicates that only a small proportion of fish move between connected caves. The variation in morphology of Arctic charr in our study likely partially results from both temporal and spatial differences in food availability. For instance, the growth of Arctic charr varies among years and within years (summer/ winter months) in this system (Mittell et al., in prep) indicating clear differences in resource availability, as seen in other spring fed systems [41]. However, this will require further studies.

In the caves fish have two potential sources of prey: aquatic invertebrates and terrestrial insects. The caves vary in the quantity of aquatic invertebrates both on the benthos and in the epibenthic area (Kristjánsson et al. in prep), which are known food for small Arctic charr [34, 41]. The density of invertebrates and their communities in these caves are known to respond to environmental variables, especially the amount of available energy, and to some extent pH (Kristjánsson et al. in prep). The second prey source is the external input from the periodically emergence of chironomid midges from Lake Mývatn. These are both spatially and temporally variable food for the fish [36, 42–44]. These midge swarms vary considerably within each year and are mostly seen in May/June and then in August. At the same time, they are also highly variable among years. The amount of flying adults, and carcasses of midges that deposit at the surface of the water, also likely varies among caves due to geographic distance from the lake [45], and due to the area of water in the caves exposed to the sky. Prey availability - such as the effects of midge abundance and timing of emergence -, have the potential to alter fish phenotypes with direct effect on growth and feeding preferences of individuals as seen in temperature-stable spring system [41]. Other factors, such as the number of openings per cave, the size of the cave and their orientation and exposure may be linked to productivity, and may affect important functional traits such as trophic structures and overall head shape. Future work on spatial and temporal fluctuations of prey availability, and a finer characterisation of local habitats (in space and time) is needed to better understand morphological divergence of these small populations, especially in the absence of strong environmental gradients.

### Environment versus neutral processes shaping phenotypic divergence

The mixed modelling approach revealed that temperature, the distance to the lake and the number of cave openings explained only a small amount of the variation in fish shape among populations. The proportion of phenotypic variance explained by those environmental factors was in two (out of six) instances higher than that of the genetic contribution (40% of the phenotypic variance among caves in head morphology PCA2_head2_ and 30% in body shape PCA3_body_ – Fig. 6). This indicates that the measured parameters (temperature, distance and cave opening), or other correlated ecological parameters, influence phenotypic divergence between the caves. Interestingly, this approach also identified and quantified other parameters not included in the model that may substantially contribute to phenotypic variance among caves (PCA1_head_ and PCA3_body_). Data on food availability and prey assemblages would be important factors to be included in the model, in addition to abiotic parameters that are known to drive differences in fish morphology - such as dissolved oxygen, light availability or type of substrates (e.g. [46, 47]).

Alternative explanation for the non-matching of the genetic and phenotypic clusters of these populations could be that even under similar environmental pressures some populations show unique responses resulting from population specific ecological and evolutionary histories [48–51]. Some of the populations had very low population sizes that were well below the believed viable minimum in a population [52]. However, there were larger populations found in larger caves where populations size may be comparable to small fish populations previously studied [18, 20]. We do not know how well these populations will be able to evolve in relation to variation in ecology (evolvability), but it likely varies with the range of population sizes observed in the caves, where in smaller population you can expect that genetic diversity is mostly the product of neutral processes while larger populations may have higher level of genetic variation and therefore higher evolvability. However, recent work suggests that even small populations with low genetic diversity can respond to selection [53, 54], and constitute a reservoir of genetic and phenotypic variation adapted to local ecology [20]. In small populations, quantitative genetic variation, which is the target of natural selection, may be retained through environmental stochasticity [55], purging [56], biased selection of more heterozygous individuals [57–59], and low levels of immigration who will be sufficient source of gene flow to lift inbreeding risks [60–62]. Our study system, with the ongoing longitudinal tracking of individuals across the replicated set of cave populations and fine scale ecological monitoring, will contribute to teasing apart ecological and evolutionary mechanisms at play in small populations.

## Conclusions

We documented neutral genetic and phenotypic variation of 24 small recently diverged populations of Arctic charr. The unique and pristine setting of these lava cave populations combined with the high degree of diversity known in Arctic charr provides a unique set up to study adaptive divergence of young small populations in the wild. Although the relevance and the finesse of the environmental metrics might be specific to this study system, if comparable metrics are combined with genetic data they can increase our power to detect signature of selection or plasticity even in populations found over a small spatial scale and/or in populations that show subtle environmental differences. Here, we showed that populations with varying degree of physical connectivity and population sizes can display substantial phenotypic divergence, even at a small spatial scale. The spatial pattern of phenotypes appeared to be mostly driven by neutral processes as classically expected in small populations [61], or derived from phenotypic composition of founding populations. However, our data also suggests that some amount of phenotypic variation may be explained by local environmental factors, highlighting the importance of collecting environmental data as well as genetic and phenotypic data.

Teasing apart the effect of local ecology in small populations found over a small spatial scale can be difficult as ecological gradients may not be pronounced. Although we did not measure selection or the genetic basis of phenotypic traits, the fact that neutral processes did not explain entirely the pattern of phenotypic variation yield promises in understanding how neutral evolutionary processes can interact with other forces of selection at early stages of divergence. The accessibility of these populations has allowed us to establish a long-term monitoring of individuals in 20 of these caves which will deepen our understanding of how the environment (through selection or plasticity) can influence phenotypic and genetic divergence, in shaping and maintaining diversity in small populations.

Such studies are important, especially in freshwater, as habitat alteration is commonly breaking populations into smaller units, which may or may not be viable. Finally, following multiple populations for demographic traits – such as population sizes and age class composition- and standing genetic variation, and how they interact with the changes in the environment are essential to better predict response to climate change.

## Methods

### Study site

This study was conducted in 24 lava caves next to lake Mývatn, North East Iceland (65°36′ N, 17°00 W). The lake and the lava caves were formed about 2300 years ago following a major volcanic eruption [63, 64]. In the lake, two morphs of Arctic charr have been described: a generalist morph and a benthic morph (local name Krús), the latter found in the south-eastern part of the lake where cold groundwater emerges [65]. The caves are located on the western side of the lake and it is assumed that individuals from either or both of these morphs (or their ancestors) may have founded the cave charr populations.

The landscape around the lake is dominated by lava features, such as pillars, pseudo craters and caves. The lava caves are internal structures in a pahoehoe lava sheet, created by a reduction of the volume of molten lava under a solidified crust. The reduction in volume may have been due to changes in flow dynamics of the molten part, its degassing, or both [66]. The lava originated in an eruption in the period 350–170 BCE [67] which also created Lake Mývatn [68].

The lava caves were created from air pockets trapped under a thin lava layer, which subsequently collapsed after the lava cooled. Therefore, caves can have multiple openings, and the exposure of the water surface to the open sky varies, and in some cases caves can have underground connections. Our study included caves in the Haganes area (West of the lake) and the Vindbelgur area (North West of the lake) (Fig. 1). The Haganes (H) caves can be subdivided geographically into Northern (H-N), Central (H-C) and Southern (H-S) subareas, while the Vindbelgur (V) area caves are divided in Eastern (V-E) and Western (V-W) subareas. Previous studies have identified 329 caves openings in H and 44 in V area (Árni Einarsson, unpubl. data). Many of the caves contain groundwater fed ponds, where small Arctic charr have been observed into approximately half of these openings (Árni Einarsson, unpubl. data). These ponds are shallow, commonly below 1 m in depth with max depth likely not more than 3 m. In addition, threespine stickleback (*Gasterosteus aculeatus*) hass been observed in a few cave, and nearby ponds [69].

### Cave selection and fish sampling

We selected 24 caves across H and V areas (15 and 9 respectively) based on the following criteria: [1] fish were observed (during an initial 10-minute observation period), and (2) the cave was amenable to sampling by trapping and electrofishing (e.g., sufficient height of ceiling, width of the cave, water depth).

We have monitored the caves in June and August, with each cave being sampled twice during both the June and August sampling periods, with about a week interval. The sampling started in 2012, and has continued since then. The environment of each cave was documented by measuring ecological and geomorphological data. We placed HOBO data loggers (UA − 001-64 and UA-002-64 Onset Corporation) in each cave for 3 years (2012–2014) measuring four times during the day and estimated average annual temperature (to the nearest 0.1 °C). We counted the number of openings for each cave as a proxy for cave size: larger caves had more openings. We measured the size of each opening and the amount of water exposed to air (m^2^) using standardized transects. The size of the caves, the number and the size of openings is a proxy for prey diversity (terrestrial food sources and the diversity and density of the invertebrate community in each cave, Kristjánsson et al. in prep). We also estimated the minimum linear distance (in meters) of each cave to the closest edge of the lake using GPS coordinates.

For studying the morphology and the allelic composition of the cave charr we sampled fish in June and August 2012, when five to ten un-baited minnow traps (depending on the size of the cave pond), were laid near the opening(s) and left overnight (Fig. 2). The traps were removed the following day and checked for the presence of fish. The cave was then intensively electrofished to increase capture rates. We caught 973 individuals, used for morphometric analysis and genotyping (Table 1). Our data showed that some cave populations of Arctic charr are connected (fish movement observed between pairs of caves, see below), but each cave was treated as a separate population in subsequent analyses.

Arctic charr from Lake Mývatn (average depth 2.5 m) were collected to compare morphology and the genetic composition of the cave charr to the putative ancestral lake populations. We collected 49 fish of the benthic morph (Krús) using electrofishing along the shore at Syðrivogar, where coldwater springs enter the lake (Table 1, Fig. 1). Those fish were processed in a similar way as the cave charr. Allelic composition of the generalist morph found in the lake was obtained from 50 fin clips collected in 2012 by the Marine and Freshwater Institute (Hafrannsóknarstofnun, Rannsókna- og ráðgjafastofnu hafs og vatna) as part of their yearly monitoring project [65]. No information on the morphology of these fish was available, therefore, the generalist morph was included in the genetic but not the morphological part of this study.

### Phenotypic, genetic sampling and population size estimation

Upon capture, each individual was anesthetized with phenoxyethanol 300 ppm for phenotyping and marking. Each fish was weighed to the nearest 0.01 g. Subsequently, the individual was placed on a measuring board with a mm scale, its fork length (length from the snout to the anterior fork of the caudal fin) measured (to the nearest 0.1 cm) and its left side photographed using a digital camera (Cannon 650D and f.1.8 50 mm fixed lens). A small portion of the upper half of the caudal fin was cut with sharp surgical scissors for genetic analysis. After fin clipping and tagging (see below), the fish were placed in a bucket of fresh water until they recovered from anaesthesia and released back to the cave they were sampled from. The tissue samples were preserved in 96% ethanol, which was replaced within 48 hours, and kept frozen at − 20 °C until DNA extraction.

To allow individual identification of the fish, we used different tagging methods depending on the size of the fish. In June 2012, fish above 45 mm were marked by a cut to their upper caudal fin so that they could be re-identified, based on clear scar tissue, in August as “recapture” (i.e. to avoid sampling the same fish twice). In August 2012, fish 65 mm and above were tagged using 12 mm PIT tags (HDX; Oregon RFID), and fish from 45 to 64 mm were tagged with color Visible Implant Elastomer tags (VIE; Northwest Marine Technology, WA, USA). For PIT tagging, a small incision (< 2 mm) was made with a sharp surgical scalpel in the anterior body cavity and the PIT tag manually inserted. VIE tags were injected with a syringe under the skin at different locations (the base of the dorsal, anal and/or caudal fins) to allow individual identification. Our tagging method followed approved standard procedures for similar sized salmonids [70]. Five individuals died during capture, sample collection, and tagging (i.e. 0.9% mortality rate).

Census population size was estimated with the Lincoln-Petersen method [71] based on mark-recapture data from 2012 to 2014. From the 24 caves initially sampled in 2012, two caves were not sampled in 2013 and 2014, as they were not accessible enough for our standardized electrofishing methods.

### Geometric morphometrics

We investigated the fine scale phenotypic diversity of the cave populations and the lake morph Krús using geometric morphometrics. We digitized 21 landmarks (15 fixed and 6 sliding semi landmarks) from the digital images of each fish (Appendix 8) using tpsDig2 from the tps program series [72]. The morphometric data were corrected for up—or down—bending of the specimens using the “unbend” module in the tpsUtil program. We conducted two separate analyses: (1) one on body shape (21 landmarks) and (2) one on head shape by selecting a subset of landmarks (1 and 13–21; Appendix 8). We expected body shape to be more seasonally variable (i.e. labile) reflecting the body condition of the fish (i.e. depth of the body behind the opercula), while the head shape would be a more robust shape measure and describe functionally relevant traits connected to feeding morphology.

We characterized morphological variation using the R package *geomorph* [73]. Generalized procrustes analysis was conducted to remove the isometric effects of size on shape, rotation, and translation from all specimens simultaneously. The analysis returns shape information, aligned Procrustes coordinates, and centroid size. Centroid size is a measure of the overall size of each fish and is calculated as the square root of the sum of squared distances of all the landmarks from their centroid. Centroid size is related to the length of the fish (here: fork length FL), but also to the thickness of the body and other indicators of condition. We examined the association between centroid size and fork length (FL) of all the fish using a linear regression and found the relationship to be weak (*P* < 0.05, R^2^ of 1.5% of variation). This indicates that centroid size may be more related to fish condition than fish length. FL was therefore used in the subsequent analyses to assess the effect of allometry on shape.

Using Procrustes linear models, we tested for significant differences among populations in morphology, with FL as a covariate. In these models, we also tested for homogeny of allometric relationships among populations, by examining the interaction of FL and cave population. The analysis revealed significant interaction terms indicating that allometric relationships existed among populations and were an important component of the morphological variation. Therefore, we did not remove the allometric effect of FL prior to further analyses, but are aware that the morphological differences among populations include allometric effects on shape.

To assess morphological differences in body and head shape, we used two multivariate analyses i) a Principal Component Analysis in the *geomorph* package and ii) a linear discriminant Analysis (DFA) in the *MASS* package [74] with caves as a grouping variable. The PCA analysis summarized the information of variations and mean shapes based on the landmarks. The DFA analysis is a classification test and was used here to (1) maximize the differences in shape among populations and to check (2) the accuracy of classification to a priori population based on shape.

We calculated pairwise morphological distances across the caves using the function *morphol.disparity*. This function returned the dissimilarities or morphological distances between the average morphology of fish in each pair of caves. We then tested whether morphological distance in body shape was related to geographic distance using a Mantel and a partial Mantel test (Ecodist package [75]), to account for geographic clustering in the same way as with the genetic data.

### Genetic analyses

- *Microsatellite genotyping*

Genomic DNA was isolated from 1072 tissue samples (973 cave, 49 Krús and 50 generalist Arctic charr) using a phenol-chloroform protocol (modified from [76]). This extraction method was used because the yield and quality of DNA for small tissue samples were better than for commercially available kits. We genotyped all fish for variation at nine microsatellite loci: OMM1236 (GenBank: AF470016.1), OMM1329, OMM1302 [77], BX890355 (GenBank: BX890355.3), Omi179TUF (GenBank: AB105856.1), OMM1228 (GenBank: AF470009.1), OMM5151, OMM5146 [78], OMM1211 (GenBank: AF469995.1).

Polymerase chain reactions (PCR) were performed in 10 μl reaction mixtures, which contained 3 μof 15 ng/ul DNA, 1 x buffer, 1.2–1.5 mM MgCl2, 0.1 mg/ml BSA, 0.2 mM dNTP, 0.15 μM of fluorescent labeled primer, 0.2 μM forward primer, 0.6 μM reverse primer, and 0.041 U/μl Taq DNA polymerase using a similar PCR program as described in [79] but adjusted to 35 cycles. Loci were amplified individually using locus-specific annealing temperatures and the forward primer for each marker was fluorescently labelled for subsequent visualization (M13). Four fluorophores (6FAM, NED, PET and VIC) were used so there were two to three markers with different allele sizes per dye. The same dye was always used for a given locus as to avoid genotyping error linked to dye-shift [80]. Genotyping was done with an automated system where the products from the nine PCR reactions were pooled and separated using ABI 3770 DNA Analyzer and visualized using Peak Scanner™ Software (Applied Biosystems). Fragments were sized using a 350-TAMRA size standard.

(b) *Genetic diversity*

We calculated basic descriptive statistics of allelic variation, including the number of alleles (N_A_), observed (H_O_) and expected heterozygosity (H_E_) at each locus and in each population, using MSA [81]. Allelic richness (A_R_) was calculated using the rarefaction method as implemented in FSTAT version 2.9.3.2 [82]. All loci in each population were checked for the presence of null alleles using the microsatellite analyser MICROCHECKER 2.2.3 [83]. We tested for deviation from Hardy-Weinberg proportions at all microsatellite loci using exact tests implemented in GENEPOP version 4.0 [84]. Critical significance levels for all relevant tests were adjusted using sequential Bonferroni correction [43].

(c) *Population genetic structure*

Heterogeneity in allele frequencies between all population pairs (24 caves and two lake samples) was determined with Fisher’s exact test using FSTAT [82]. We also examined the distribution of genetic variation within and among populations with *F*-statistics. Population differentiation was considered significant if the confidence interval for the multilocus estimate of *F*_*ST*_ [44] based on 1000 data permutations, excluded zero. We also calculated the ‘actual differentiation’ estimator *D*_*est*_ [85] using SMODG 1.2.5 [86] as it avoids the inter-relationship of *F*_*ST*_ with the amount of polymorphism within populations [87].

The genetic affinity of the 24 cave and two lake populations to each other was evaluated using two approaches. First, pairwise values of Cavalli-Sforza and Edward’s chord distance (*D*_*ce*_) were used to construct a consensus neighbour joining phenogram using PHYLIP 3.5 [88]. One thousand bootstrap replicates were used to determine the statistical support for each node. We used *D*_*ce*_ rather than *D*_*a*_ because it is more likely to achieve the correct tree topology. A parallel analysis with *D*_*a*_ resulted in an identical tree topology (data not shown). The small sample sizes for some caves did not impact the results as repeating the analysis after excluding populations with *N* < 20 returned the same tree topology. Second, we investigated the genetic structure of the 24 cave and two lake populations independent of geographic sampling by the Bayesian statistical framework implemented in the software STRUCTURE 2.3.3 [89, 90]. A burn-in period of 50,000 iterations and a sampling period of 150,000 iterations in admixture models (where a fraction of the genome of each individual is equally likely to originate from each population under consideration) were used. We performed runs for 1 to 30 clusters (K, the putative number of biological populations) with 15 iterations for each K to quantify the variation in likelihood, and calculated the logarithm of the mean posterior probability of the data L(K). To identify the most likely number of K, we used the maximal value of L(K) returned by STRUCTURE (e.g. [91]) and calculated the DK statistic using the second-order rate of change in log probability between successive K values [92] using the program Structure Harvester [57]. Clumpack software [58] was used to create a bar plot of membership proportions for all individuals. The STRUCTURE analysis was rerun after excluding populations with *N* < 20 and returned the same result as the full analysis.

Because these populations of Arctic charr appear to be landlocked with limited dispersal ability, we tested whether the patterns of neutral genetic structure were the result of isolation by distance (i.e. stronger divergence across larger geographic scales due to reduced gene flow and increased role of drift). For the isolation by distance calculation, we used *Dst/(1-Dst)* as *F*_*ST*_ has been reported to be sensitive to small sample sizes [59]. Geographic distances were calculated as linear distances (in km) between the GPS coordinates of the caves from each other or from the lake. We tested whether genetic distance was related to geographic distance using a Mantel test implemented in the *Ecodist* package. We also ran a partial Mantel test to control for the effect of geographical area (H, V and the two morphs in the Lake) on the relationships between distance matrices. This involved using a coding variable that identified the geographical areas included in a given pairwise comparison.

### Partitioning of environmental effects and neutral processes on phenotypes

In order to estimate phenotypic variation associated with (i) environmental variation, (ii) genetic structure among caves, and (iii) residual among-cave variance (i.e. neither explained by available environmental variables nor associated with genetic relationships among caves), we conducted mixed model analyses. In these models, we used individual body and head shape principal component scores (respectively PC_body_1, 2, and 3 and PC_head_1, 3 and 5) as response variables, and environmental parameters as fixed effects. The Cavalli-Sforza and Edwards chord distance matrix (*Dce*) was used to structure the covariance of random effects (see eq. 2) describing similarity among caves that is associated with genetic structure. The models took the form.

1 $$E{\left[z\right]}_{ij}=\alpha +{\beta}_L{L}_i+{\beta}_T\ {T}_j+{\beta}_O{O}_j+{\beta}_D\ {D}_j+{\delta}_T{T}_j{L}_i+{\delta}_O{O}_j{L}_i+{\delta}_D{D}_j{L}_i$$

2 $${z}_{ij}=E{\left[z\right]}_{ij}+{g}_{a,j}+{g}_{b,j}{L}_i+{c}_{a,j}+{c}_{b,j}{L}_i+{e}_{i,j}$$

Equation 1 specifies the fixed effects component of the mixed model, wherein expected values of phenotype *z* of individual *i* in cave *j* are described as a function of individual fork length (FL) (L_*i*_), and cave-specific values of temperature (T_*j*_), area of openings (O_*j*_) and distance from the lake (D_*j*_), as well as interactions between FL and the cave-specific environmental variables. The model intercept is denoted *α*, partial slopes describing the average effects of length, and environmental variables on phenotype are denoted *β*_*L*_, *β*_*T*_ , *β*_*O*_, and *β*_*D*_, for effects of FL, temperature, area of the openings, and distance from the lake. Corresponding interaction terms, describing how the effect of FL on morphology may differ according to ecological variables are denoted *δ*_T_, *δ*_O,_ and *δ*_D_. All covariates were mean-centered and scaled to unit variance prior to analysis. As such, the regression coeffients (*β* terms) can be interpreted as the average partial effects of each cave-specific variable, for individuals of average length.

Equation 2 represents the random regression component of the mixed model, wherein variance not explained by the fixed effects is described by random intercepts and slopes for each cave, describing associations with the genetic structure, and variance that is independent of the genetic structure. The key parameters of interest are the variances of intercepts associated with genetic differences (*g*_*a*, *j*_) and independent of genetic differences (*c*_*a*, *j*_). Random slopes are not of direct interest but are included to allow for the possibility that relationships between phenotype and length may differ among caves, in addition to variation in slopes described by the fixed interaction terms.

### Proportions of among-cave variance associated with environmental variables and genetic structure

While the variances of random intercepts associated with the genetic structure, and variance not associated with the genetic structure, can be used directly in the partitioning of phenotypic variance among caves, the variance attributable to the environmental variables, which are treated as fixed effects, are not directly returned by the mixed models. However, de Villemereuil et al. [93] described how to recover the variance in phenotype associated with fixed effects, given the variance of the fixed predictor variables (in the present case, the environmental variables), and the estimated effects of the predictors on the response (on phenotype). In our analysis the variances of phenotypes associated with the cave-specific environmental variables are given by.

3 $${\sigma}_e^2={\beta}^t{\varepsilon}_E\ \beta$$

where Ɛ_*E*_ is the covariance matrix of the cave-specific environmental variables: temperature, opening area, and distance from the lake. *β* is a vector of the partial effects of each variable on phenotype, modelled as *β*_*T*_, *β*_*O*_, and *β*_*D*_, in eq. 1.

To characterise different aspects of phenotypic repeatability at the cave level, we first calculated repeatabilities of morphology at the cave level, in relation to the total variation among individuals. The total variance among caves, isometrically and allometrically independent of FL, is thus ${\sigma}_t^2$ = ${\sigma}_e^2$ + ${\sigma}_g^2$ + ${\sigma}_c^2$. The total variance among individuals is ${\sigma}_T^2$ = ${\sigma}_t^2$ + ${\sigma}_e^2$, where ${\sigma}_e^2$ is the mixed model residual variance.

We then further decomposed the variation in morphology among caves into components associated with the environmental variables, a proportion that is associated with the genetic structure of the charr in the caves, and finally a proportion unexplained by genetics and the available environmental variables. The proportion of variance associated with the environmental variables (E), the genetic structure (G), and other source of variance (O) explained were calculated as $\frac{\sigma_e^2}{\sigma_t^2}$, $\frac{\sigma_g^2}{\sigma_t^2}$, and $\frac{\sigma_c^2}{\sigma_t^2}$, respectively. Finally, we calculated the proportion of the total variance among caves, conditioning on the environmental fixed variables, (i.e., ${\sigma}_g^2$ + ${\sigma}_c^2$) and expressed it as a proportion of the total variance, including variation associated with measured environmental differences, i.e. of ${\sigma}_t^2$.

All statistical analyses were performed in R version 4.0.2 [94].

### Supplementary Information

**Supplementary Material 1.**

## Data Availability

The datasets used and analysed in this study are available from the corresponding author on reasonable request.
