# Supplementary material for: Fine scale diversity in the lava: genetic and phenotypic diversity in small populations of Arctic charr Salvelinus alpinus
Source: BMC Ecol Evol. 2024 Apr 15;24:45. doi: 10.1186/s12862-024-02232-3 (PMC11017478; doi:10.1186/s12862-024-02232-3)
Supplement: Supplementary file 1 — Supplementary Material 1. [file 12862_2024_2232_MOESM1_ESM.docx]

**SUPPLEMENTARY MATERIAL**

**Appendix 1**

**Estimates of population sizes (Nc) of small Arctic charr (*Salvelinus alpinus*) in lava caves, Iceland.** Census population sizes (Nc) were estimated from multiple captures of fish between 2012 and 2014 using the Lincoln-Petersen method, and effective population sizes (Ne) were calculated from a single estimate in time (2012) using the LDNe method. The bottom lines of the table indicate estimation of population sizes in connected caves based on fish movement between caves. The total number of migrants (e.g. the number of fish that were found in another cave that their previous capture) was calculated for each pair of connected caves between 2012 and 2014.

| **Caves** | **Nc** | ***Ne*** | ***CI Ne (Jackknife on loci)*** |  |
| --- | --- | --- | --- | --- |
| 1 | 25 | 15 | (3 – 698) |  |
| 2 | 35 | 9 | (2 – 59) |  |
| 3 | 16 | 15 | (3 – inf) |  |
| 4 | 19 | 11 | (1 – inf) |  |
| 5 | 63 | 71 | (25 – inf) |  |
| 6 | 11 | 2 | (2 – 6) |  |
| 7^*^ | 96 | 169 | (54 – inf) |  |
| 8 |  | -18 | (9 – inf) |  |
| 9 |  | 82 | (14 – inf) |  |
| 10 | 35 | 28 | (11 – 203) |  |
| 11 | 88 | 124 | (40 – inf) |  |
| 12^*^ | 51 | 84 | (27 – inf) |  |
| 13 | 15 | -149 | (15 – inf) |  |
| 17 | 17 | -11 | (16 – inf) |  |
| 17b | 26 | 141 | (5 – inf) |  |
| 18 | 100 | 47 | (16 – 954) |  |
| 19 | 37 | 33 | (11 – inf) |  |
| 20 | 82 | 23 | (10 – 77) |  |
| 21^*^ | 48 | 51 | (14 – inf) |  |
| 22^*^ | 115 | 30 | (16 – 68) |  |
| 23 | 60 | 6 | (3 – 14) |  |
| 24 | 42 | 22 | (8 – 829) |  |
| 25^*^ | 412 | 186 | (94 – 687) |  |
| 26 | 126 | 22 | (13 – 39) |  |
| Lake - generalist |  | 31 | (21 – 52) |  |
| Lake - Krús |  | 214 | (63 – inf) |  |
| **Connected caves** |  |  |  | **Migrants** |
| 1 and 2 | 87 | 13 | (4 – inf) | 10 |
| 5 and 11 | 151 | 87 | (43 – inf) | 6 |
| 8 and 9 | 16 | 15 | (12 – inf) | 1 |
| 17 and 18 | 117 | 47 | (18 – inf) | 3 |
| 19 and 20 | 159 | 42 | (18 – inf) | 1 |
| 7 and 25 | 550 | 169 | (54 – inf) | 19 |

* these caves were visited 3 times during the year 2012 (once in June and twice in August) within a week interval.

Nc could not be calculated in populations that were visited only once.

Negative Ne should be interpreted as infinity and likely results from a low sample size.

*Na* in the migrants column indicates that fish calculation of migrants could not be made for this pair of caves. Caves 8 and 9 were only visited twice.

**Appendix 2**

Pairwise comparisons of genetic differentiation among populations of Arctic charr from 24 caves (C) and two populations from Lake Mývatn (L-Krús = Krús; L = generalist). D_est_ values are above the diagonal and F_st_ values are below diagonal. Fst values in italics and yellow are not significant at the 5% level after Bonferroni correction.

**Appendix 3** – Delta K values for different numbers of populations assumed (K) in the structure analysis (see figure 3B in the manuscript). Delta K was calculated as DeltaK = mean(|L''(K)|) / sd(L(K)).


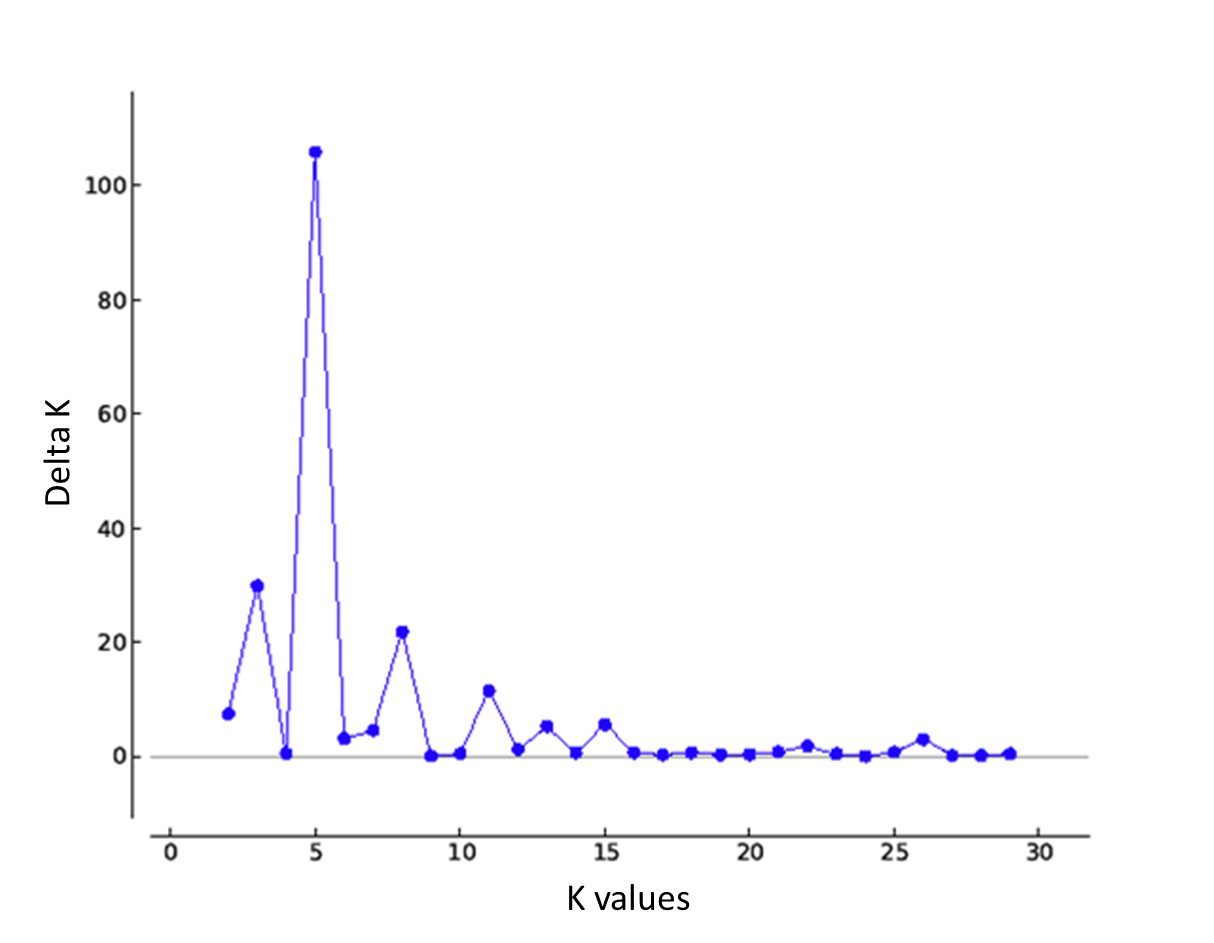


**Appendix 4**

**Principal component analysis of (A) body and (B) head shape among 24 cave populations and one lake population (Krús) of Arctic charr sampled from the Mývatn area, Iceland.** Shape analysis was conducted using landmarks-based geometric morphometric of body/head morphology. Each point represents the average body/head shape of fish in a given population. Numbers refer to the cave numbers as described in Table 1. The deformation grids show the average morphology of fish/head in a population with a 3-time magnification, at the extremes of each axis. The genetic cluster that the majority of fish in a population were assigned to is indicated by the colour of the average body shape point (see Table 1 and Figure 1 and 3 for details).

1. Body shape

(B) Head shape

**Appendix**  **5**

Correlation between the genetic and morphological distances (body shape) of the 24 lava caves and the two lake populations of small Arctic charr. Mantel and partial Mantel tests were non-significant.


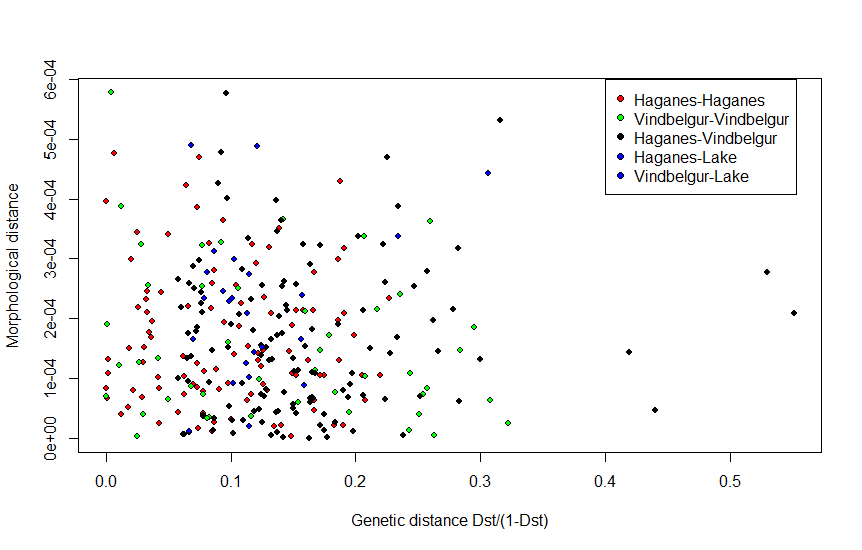


**Appendix 6**

Repeatabilities of morphology at the cave level, in relation to the total variation among individuals within each cave. Six morphological axes were considered, 3 characterising body shape (PC1, 3, and 5), and 3 axes for head shape (PC1, 2 and 3). The total variance among caves was isometrically and allometrically independent of length.


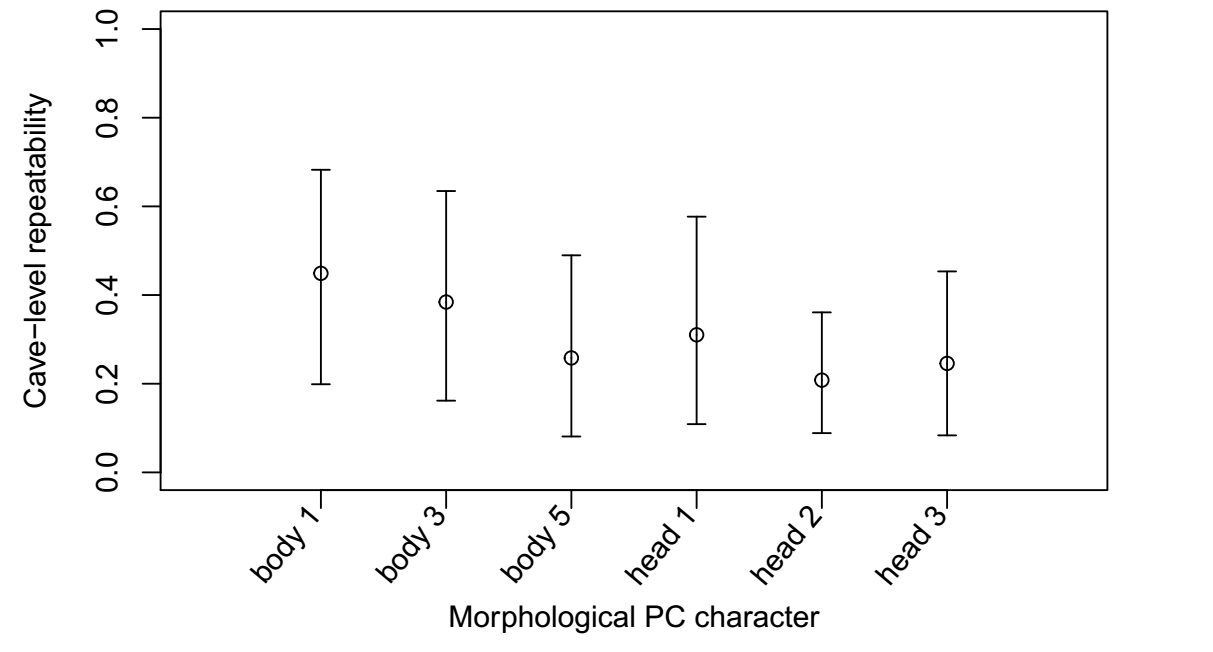


**Appendix 7:**

Results of the mixed models. Table (a) are the fixed effects estimates and table (b) are the random effect covariance estimates.

(a)


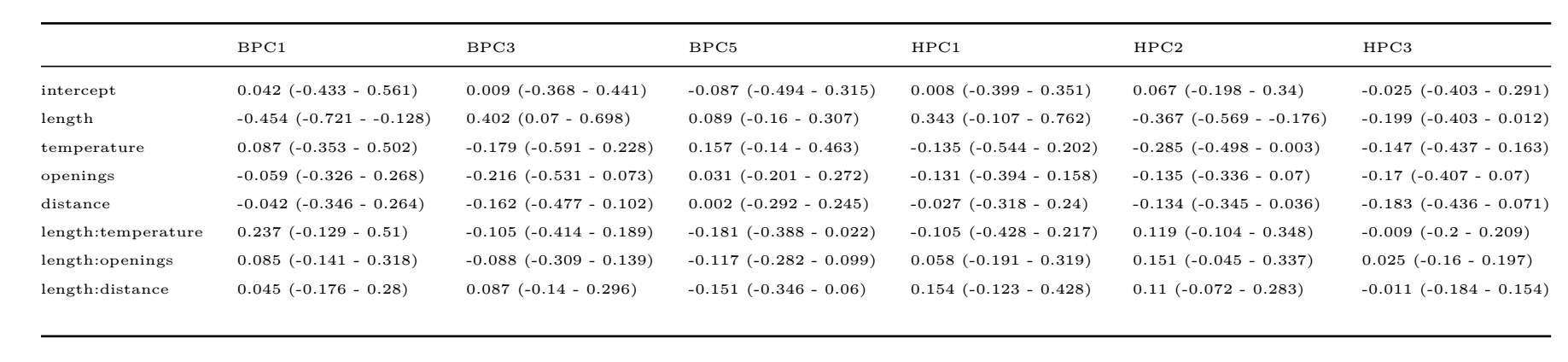


(b)


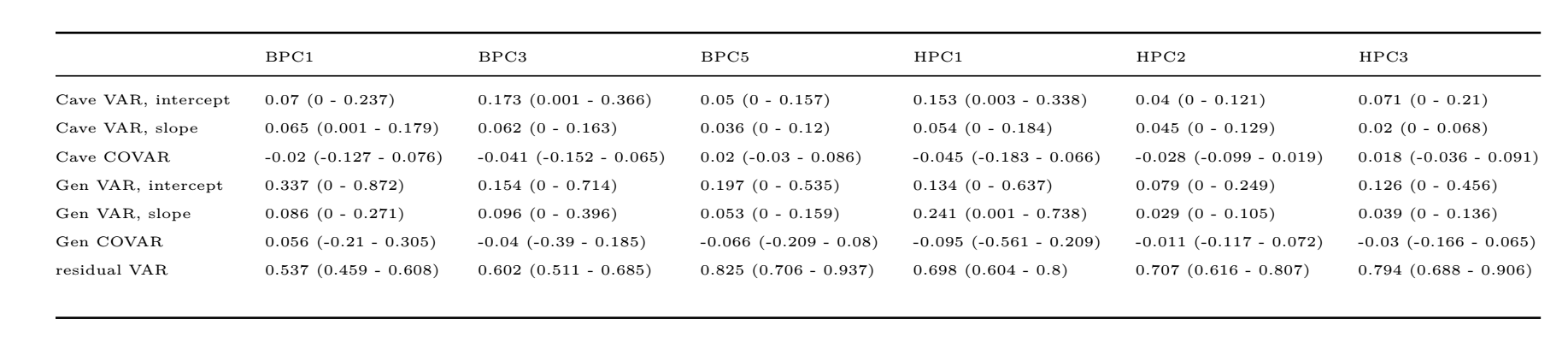


**Appendix 8**

Definitions of landmarks and sliding semi landmarks (SL) used in lateral view configuration of small Arctic charr (*Salvelinus alpinus*). Body shape characterization used all landmarks and head shape was characterized by landmark 1 and landmarks 13 - 21. (A) Photograph of the left side of a fish with all landmarks using for body and head shape analyses. (B) description of each landmark, modified from Skoglund et al. 2015; and Krisjánsson et al. 2012.

(A)


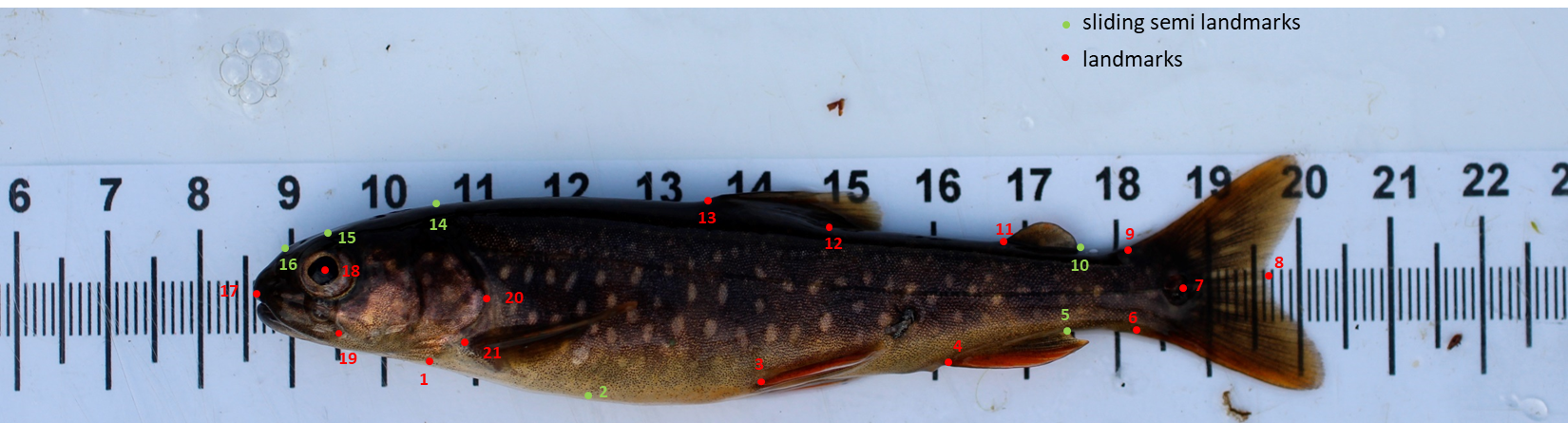


(B)

| **Landmark** | **Anatomical description** |
| --- | --- |
| 1 | Ventral point of the opercula opening |
| 2 | Half distance between landmarks 2 and 3; SL |
| 3 | Anterior insertion of the pelvic fin |
| 4 | Anterior insertion of the anal fin |
| 5 | Posterior insertion of the anal fin; SL |
| 6 | Ventral insertion of the caudal fin |
| 7 | Posterior point of the hypural bone at the lateral midline |
| 8 | Fork of the caudal fin landmark |
| 9 | Dorsal insertion of the caudal fin |
| 10 | Posterior insertion of adipose fin; SL |
| 11 | Anterior insertion of adipose fin |
| 12 | Posterior insertion of dorsal fin |
| 13 | Anterior insertion of dorsal fin |
| 14 | Posterior edge of the cranium at anterior point of the opercula; SL |
| 15 | Top of cranium at midpoint of eye; SL |
| 16 | Middle of snout; SL |
| 17 | Upper tip of the snout |
| 18 | Center of the bony orbit of the eye |
| 19 | Posterior point of the maxilla |
| 20 | The most posterior point on the curve of the operculum |
| 21 | Anterior insertion of the pectoral fin |
